# Multi-dimensional flow cytometry analysis reveals increasing changes in the systemic neutrophil compartment during seven consecutive days of endurance exercise

**DOI:** 10.1371/journal.pone.0206175

**Published:** 2018-10-30

**Authors:** Selma van Staveren, Twan ten Haaf, Margot Klöpping, Bart Hilvering, Gerjen H. Tinnevelt, Karin de Ruiter, Maria F. Piacentini, Bart Roelands, Romain Meeusen, Jos J. de Koning, Jeroen J. Jansen, Nienke Vrisekoop, Leo Koenderman

**Affiliations:** 1 Department of Respiratory Medicine, Laboratory of Translational Immunology, UMC Utrecht, Utrecht, The Netherlands; 2 TI-COAST, Amsterdam, The Netherlands; 3 Department of Human Movement Sciences, Faculty of Behavioural and Movement Sciences, Vrije Universiteit Amsterdam, MOVE Research Institute Amsterdam, Amsterdam, The Netherlands; 4 Analytical Chemistry, Institute for Molecules and Materials, Radboud University Nijmegen, Nijmegen, The Netherlands; 5 Department of Parasitology, LUMC Leiden, Leiden, The Netherlands; 6 Department of Human Movement and Sport Sciences, University of Rome “Foro Italico”, Rome, Italy; 7 Human Physiology Research Group, Vrije Universiteit Brussel, Brussels, Belgium; 8 School of Public Health, Tropical Medicine and Rehabilitation Sciences, James Cook University, Townsville, Queensland, Australia; 9 Department of Exercise and Sports Science, University of Wisconsin-La Crosse, La Crosse, WI, United States of America; University of Alabama-Birmingham, UNITED STATES

## Abstract

Endurance exercise is associated with a transient increase in neutrophil counts in the peripheral blood. Here we investigate the impact of intensified endurance exercise on the neutrophil compartment. We hypothesized that intensified endurance exercise leads to mobilization of neutrophil subsets, which are normally absent in the blood. Furthermore, we followed the potential build-up of neutrophil activation and the impact on overnight recovery of the neutrophil compartment during a seven-day cycling tour. The neutrophil compartment was studied in 28 healthy amateur cyclists participating in an eight-day strenuous cycling tour. Blood samples were taken at baseline, after 4 days and after 7 days of cycling. The neutrophil compartment was analyzed in terms of numbers and its phenotype by deep phenotyping of flow cytometry data with the multi-dimensional analysis method FLOOD. Repeated endurance exercise led to a gradual increase in total neutrophil counts over the days leading to a 1.26 fold-increase (95%CI 1.01–1.51 p = 0.0431) in the morning of day 8. Flow cytometric measurements revealed the appearance of 2 additional neutrophil subsets: CD16^bright^CD62L^dim^ and CD16^dim^CD62L^bright^. A complex change in neutrophil phenotypes was present characterized by decreased expression of both CD11b and CD62L and marked increased expression of LAIR-1, VLA-4 and CBRM1/5. The changes in expression were found on all neutrophils present in the blood. Strikingly, in strong contrast to our findings during acute inflammation evoked by LPS challenge, these neutrophils did not upregulate classical degranulation markers. In fact, our FLOOD analysis revealed that the exercise induced neutrophil phenotype did not overlap with the neutrophil subsets arising upon acute inflammation. In conclusion, during multiple days of endurance exercise the neutrophil compartment does not regain homeostasis overnight. Thereby our study supports the concept of a build-up of inflammatory cues during repeated endurance exercise training, causing a prolonged change of the systemic neutrophil compartment.

## Introduction

Peripheral blood neutrophil counts increase upon aerobic and anaerobic single exercise bouts and recover within 6–24 hours.[1] Multiple non-mutually exclusive mechanisms underlie blood neutrophilia, but little is known regarding the mechanisms of neutrophil recruitment and recovery upon prolonged exercise. A major part of the recruitment of neutrophils after at least short-term exercise is originating from the so-called marginated pool, which consists of neutrophils that are not free flowing in the blood and are (at least in part) associated with the vasculature. This pool is thought to be in complete equilibrium with the free flowing pool.[2–7] Steroids and/or epinephrine are well-known mediators that can liberate these cells from the vasculature. Steroids have been implicated in relation to neutrophilia evoked by exercise, because rises in plasma adrenocorticotropic hormone (ACTH) and cortisol levels coincide with the demargination of neutrophils.[4,8,9] In addition, treatment with high concentrations of glucocorticoids leads to neutrophilia.[10] On the other hand, stable or even decreasing levels of plasma ACTH and cortisol have also been described upon exercise. These latter studies question the hypothesis that systemic rises of these hormones are the main mechanism for the observed neutrophilia.[11–14] Furthermore, neutrophil counts can increase up to 3–4 fold upon exercise,[1] whereas only 50% of total blood neutrophils is thought to be present in the marginated pool.[3,4] Therefore, the observed increase in neutrophils does not seem to be solely explained by demargination.

Neutrophilia can also be caused by the mobilization of extra neutrophil subsets from other tissue sites (as reviewed by Pillay et al).[15] Under homeostasis peripheral blood neutrophils are homogeneous and express FcγRIII (CD16) and L-selectin (CD62L) within a narrow range, typically characterized as CD16^bright^CD62L^bright^ neutrophils. We have previously described the mobilization of two additional neutrophil subsets in response to acute inflammation such as evoked by a systemic LPS challenge in healthy volunteers. These mobilized subsets can be identified by differential expression of CD16 and of L-selectin.[16] One subset consists of CD16^dim^CD62L^bright^ cells, with predominantly band-shaped nuclei. These cells are more immature than the CD16^bright^CD62L^bright^ cells and are likely recruited from the bone marrow.[17] Interestingly, previous publications reporting neutrophilia after endurance exercise also reported a specific increase in neutrophils with a band-shaped nucleus.[18–20] Thus, one might hypothesize that this same CD16^dim^CD62L^bright^ neutrophil subset is recruited during neutrophilia evoked by exercise. The second extra subset found in the peripheral blood upon LPS challenge consists of CD16^bright^CD62L^dim^ neutrophils with mainly hypersegmented nuclei that exhibit a more activated and immunosuppressive phenotype.[16] Interestingly, this latter subset is remarkably inefficient in killing bacteria such as Staphylococcus aureus.[21] The relative increases in these subsets can have a considerable impact on the expression of the classical activation/degranulation markers CD35 and CD11b of the total neutrophil pool.[16] This possibly explains why studies staining for a single marker or a very limited number of markers after single exercise bouts have provided conflicting results.[22–24]

Currently, multi-color analysis of immune cells is the standard in medical immunology but this needs new multi-dimensional methods to adequately analyze these data. This has led to the development of data analysis tools that provide a multi-dimensional view on all markers expressed on individual cells. We have developed such a tool, FLOOD [25], that enables the recognition of multi-dimensional differences in neutrophil phenotypes by staining with many markers in addition to CD62L and CD16. Using this analysis method, we have previously reported that only normal neutrophils (CD16^bright^CD62L^bright^) are mobilized from the marginated pool upon a single anaerobic exercise bout.[25]

To get more insight into the potential role of neutrophils in the exercise-induced immune response during relatively long periods of increased training we investigated neutrophils during seven days of increased intensive endurance training. We applied multi-dimensional FLOOD analysis of peripheral blood neutrophils allowing deep phenotyping of these cells. We tested the hypothesis that the neutrophil compartment in peripheral blood would fail to recover overnight upon repetitive days of strenuous exercise.

## Materials and methods

### Subjects, study design and blood sampling

#### Tour for Life

The subjects were all healthy recreational cyclists, 11 male and 19 female aged 25–57 years (mean±SD age 40.8±10.8 y, BMI 23.5±2.1 kg/m^2^). For demographics see Table 1. At baseline the VO_2_max was 51.8±6.3 ml/kg/min and a peak power output of 4.12±0.57 W/kg was measured (performance level 2 [26,27]). Subjects reported an estimated training volume of 144±46 km per week during preparation. Written informed consent was obtained from all participants. The study was conducted in accordance with the Declaration of Helsinki and approved by the institutional ethical committee.

**Table 1:** Demographics.

Baseline characteristics for the subjects	mean ± SD
Gender (M/F)	19/11
Age (y)	40.8±10.8
BMI (kg/m^2^)	23.5±2.1
VO_2_max (ml/min/kg)	51.8±6.3
PPO (W/kg)	4.12±0.57
training volume pre TFL (km/week)	144±46

BMI: Body Mass Index; PPO: Peak Power Output

**Fig 1:**
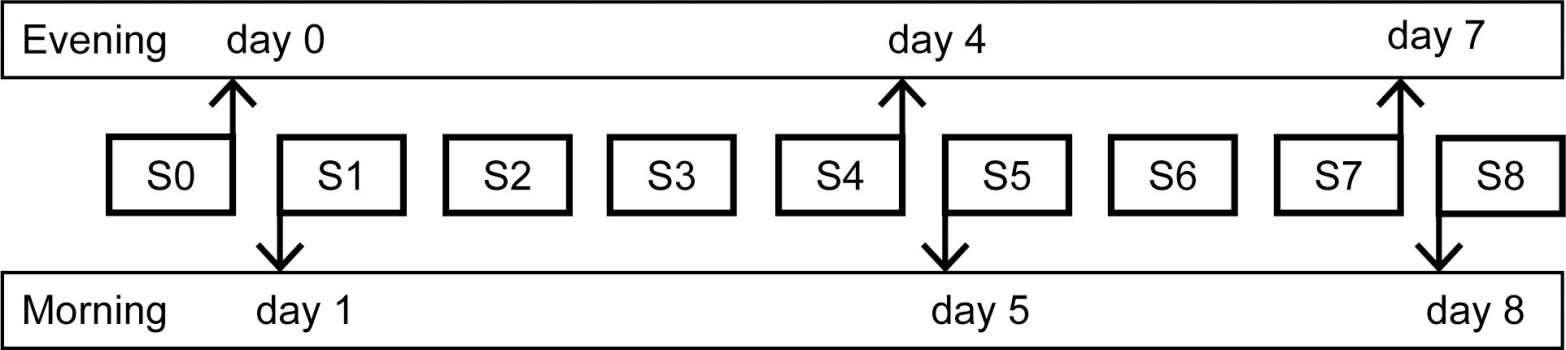
Study design. Blood was collected at 6 different time points during the TFL: before the start of the TFL in the evening of day 0 and in the morning of day 1; halfway during the TFL in the evening directly after stage 4 and in the morning before stage 5; at the end of the TFL in the evening directly after stage 7 and in the morning before stage 8. SX = Stage X.

This study was conducted during the Tour for Life (TFL). The subjects cycled from Italy to The Netherlands in 8 consecutive days (1 stage per day) together with 400 other contestants. The length, altimeters and duration per stage is shown in Table 2. The start of each stage was between 07:00h and 08:00h and the finish between 16:00h and 19:30h, depending on the fitness level of the subject and the length of the stage. At 3 evening time point and at 3 mornings during the TFL blood was sampled (Fig 1). The researchers followed the cyclists in a mobile laboratory van that enabled blood sampling, processing and freezing.

**Table 2:** Training load per stage.

Stage (day)	Distance (km)	Climbing (m)	Mean duration ± SD (min)
**1**	110	2800	387 ± 65
**2**	160	4200	592 ± 112
**3**	175	2900	564 ± 127
**4**	190	1900	531 ± 121
**5**	129	2450	420 ± 108
**6**	175	900	414 ± 60
**7**	215	2200	558 ± 83
**8**	110	1200	N.D.
**Total**	**1264**	**18550**	**3466 ± 462**

N.D.: no data available

At the mornings of day 1, day 5 and day 8 and the evenings of day 0, day 4 and day 7, blood of the contestants was collected. For cellular analyses a venous blood sample (4 ml) was collected at each occasion by venipuncture from an antecubital vein in a vacutainer tube (BD Vacutainer) containing sodium heparin as anticoagulant. For hormonal analysis at each occasion venous blood samples were collected in an EDTA vacutainer tube (3 ml, VenoSafe) and in a serum tube containing gel and clot activator (8 ml, Venosafe).

#### Experimental human endotoxemia model

Experiments were part of a larger endotoxin trial (NCT02629874 at www.clinicaltrials.gov). Healthy male volunteers 19–24 years old were enrolled after screening and were prehydrated from t = -1 h to t = 0 (2.5% glucose/ 0.45% saline at a continuous rate of 1.5 L/h) through a cannula placed in an antecubital vein. U.S. Reference E. coli endotoxin (O:113, NIH Pharmaceutical Development Section, Bethesda, MD, USA) was used as systemic challenge in this study. Endotoxin was reconstituted in 5 ml saline and injected as a single intravenous dose of 2 ng/kg during 1 min at t = 0. After LPS injection, subjects were infused with hydration fluid at a continuous rate of 150 mL/h. Heart rate and blood pressure of the volunteers were monitored from t = -1 h until discharge at t = 8 h, as well as the course of LPS-induced symptoms.[28] The study protocol was approved by the Ethics Committee of the Radboud University Nijmegen Medical Centre, The Netherlands and complies with the Declaration of Helsinki and ICH Good Clinical Practice guidelines. Written informed consent was obtained from all participants.

At t = 180 min blood samples, anticoagulated with sodium heparin, were taken from the arterial catheter.

### Cell counts and flow cytometry

For the TFL samples as well as the LPS samples, 1 ml of sodium heparin blood was analyzed by a field applicable method that has been previously described in detail.[29] In Short, 1 ml of blood was diluted in 20 ml 1:10 FACS lysing solution (Becton Dickenson, Mountain View, CA) and incubated at room temperature for 15 min for red blood cell lysis and simultaneous leukocyte fixation. The samples were centrifuged at 430 x g for 5 min at room temperature. The cells were washed twice with RPMI + 10%FCS. After the last washing step, the cells were transferred to a -30°C freezer in RPMI + 10%FCS + 10%DMSO.[29] Ten days after collection and freezing, the samples were transferred to a -80°C freezer. At the time of analysis the cells were thawed by adding 1 ml RPMI + 10%FCS at room temperature. The samples were centrifuged 430 x g for 5 min at 4°C and the cells were re-suspended in PBS + 0.32% sodium citrate + albumin (4 g/l). Cell counts were determined by an automated cell counter (CellDyn Emerald, Abbott Diagnostics). Per sample 500.000 cells were stained with a combination of 10 monoclonal antibodies and measured on a BD-LSR Fortessa flow cytometer (Becton Dickenson, Mountain View, CA). The antibodies used were as follows: CD35-FITC (clone E11), CD64-APC (clone 10,1), CBRM1/5-Alexa Fluor 700 (clone CBRM1/5), CD11b-APC-Alexa Fluor 750 (clone Bear1), CD305 (LAIR-1)-PE (clone DX26), CD14-eF450 (clone 61D3), CD16-Krome Orange (clone 3G8), CD62L-BV650 (clone DREG 56), CD49d-PECy7 (clone G9F10), CD66b-PerCPCy5.5 (clone G10F5).

### Hormonal measurements

Peripheral blood collected in EDTA tubes was centrifuged 1500 x g for 10 min and the plasma fraction was frozen at -30°C. After 8 days all plasma samples were transferred to a -80°C freezer until further analysis. Plasma ACTH concentrations were measured using an electrochemiluminescence immunoassay on the Cobas E411 (Roche Diagnostics GmbH, D-68298 Mannheim, Germany). The lower limit of detection was 2.0 ng/L and inter-assay variation was 5,7–3.5% at 8.5–170 ng/L respectively (n = 18). Intra-assay variation was <1.2%. Normative values: morning measurements 5–70 ng/L; afternoon measurements < 50 ng/L.

The serum tubes containing blood were directly incubated at 4°C for 30 min to allow clotting. This was followed by centrifugation 1500g for 10 min. The serum fraction was frozen at -30°C within 1 hour after blood collection. After 8 days all serum samples were transferred to a -80°C freezer until further analysis. Serum concentrations of cortisol were determined by a chemiluminescence immunoassay on a Unicel Dxl 800 (Beckman Coulter, Bea, CA, USA). Inter-assay variation was 5.4–8.2% at 0.50–1.05 μmol/L, respectively. Intra-assay variation 3.4–6.2% at 0.50–1.05 μmol/L, respectively. Normative values: morning measurements 0.20–0.65 μmol/L; afternoon measurements 0.10–0.35 μmol/L.

### Statistics

Two subjects were excluded from statistical analyses due to missing data on 1 or 2 time points, we used a one-way repeated-measures ANOVA test (GraphPad Prism 6.0). From the ANOVA results, we calculated fold-changes and their 95% CI to determine the statistical significance of the differences observed between time points.

The only exception was the statistical analysis of the MFI’s of the three neutrophil subsets over time, for this analysis a two-way repeated-measures ANOVA test (GraphPad Prism 6.0) was used.

### Multi-dimensional flow cytometry analysis by FLOOD

For multi-dimensional analysis of the flow cytometry data the PCA based method FLOOD was used.[25] The neutrophils were identified from the original fcs files based on FSC/SSC and CD16+CD66b+ expression (S1 Fig) (FlowJo analysis software, Tree Star Inc., Ashland, Oregon). Only cells within this gate were exported as fcs3.0 files. These files were imported into the FLOOD software.[25] Two FLOOD analyses were performed. For both analyses the control group consisted of all morning day 1 samples. For the first analysis the evening day 0, evening day 4, morning day 5, evening day 7 and morning day 8 time points were labeled as response group. For the second analysis the response group consisted of evening day 0, evening day 4, morning day 5, evening day 7 and morning day 8 time points as well as the LPS samples. A value equal to the most negative intensity -1 was subtracted, resulting in values of >1, to deal with negative intensities as a result of background subtraction by the flow cytometry software and as a result of compensation. Preprocessing steps consisted of log transformation, mean centering based on the control group and scaling based on controls. For further details on the approach we refer the reader to the original manuscript. [25]

## Results

### Neutrophil counts did not recover to baseline after 7 days of intensified endurance exercise

**Fig 2:**
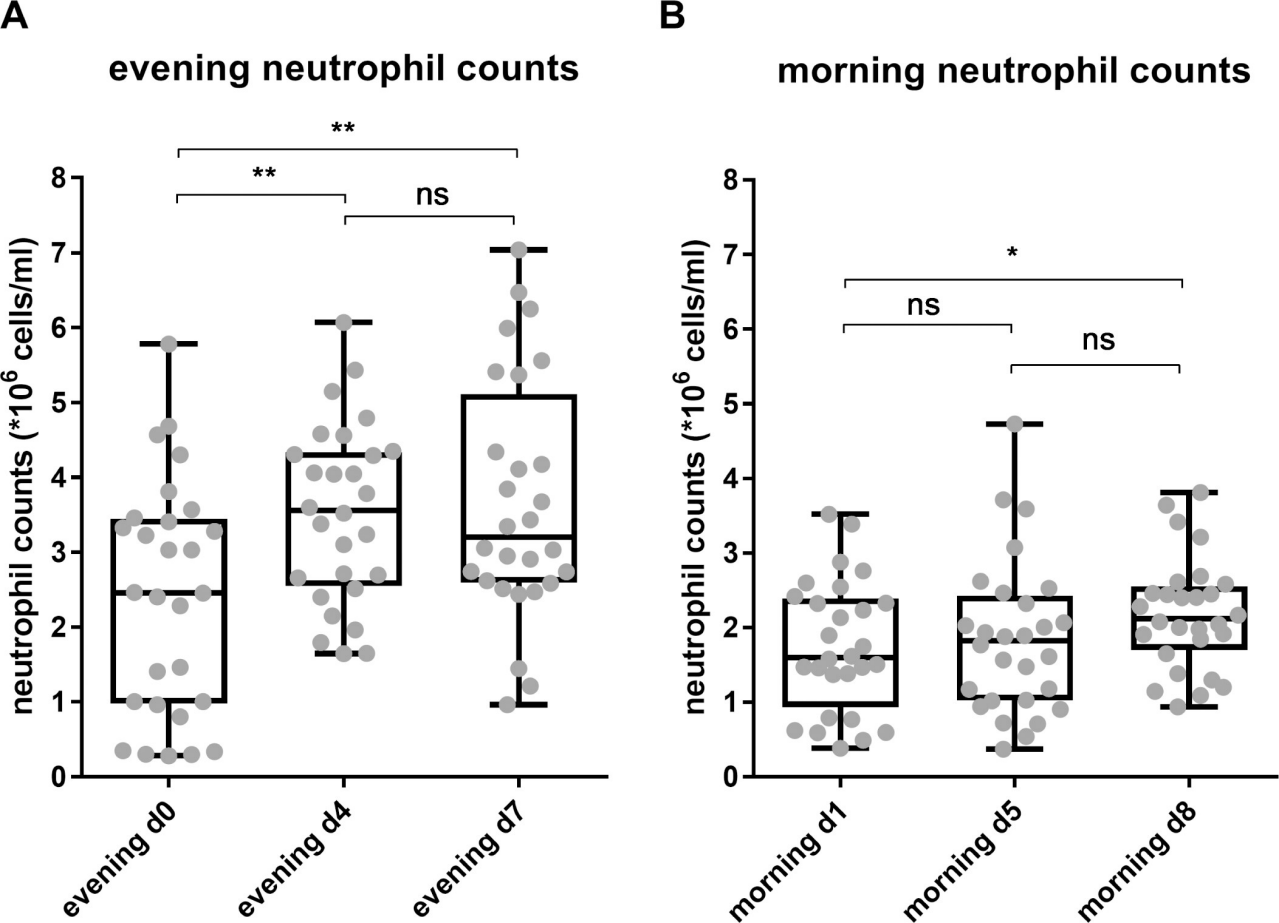
Total peripheral blood neutrophil counts increased upon daily endurance exercise and did not recover to baseline overnight after 7 days of cycling. Neutrophil counts were measured at (A) 3 evening time points and (B) 3 morning time points during the TFL. * p = 0.0431; ** p<0.005; ns = not significant.

Neutrophil kinetics were studied in 30 healthy amateur cyclists during seven days strenuous cycling (mean daily distance of 160 km and 2300 altimeters). The exercise volume of the contestants increased on average with 770 ± 24% during the week of exercise compared to their regular training schedule. We observed an exercise-induced increase in neutrophil counts in the evening of day 4 and day 7 when compared to evening day 0 (Table 3, Fig 2A). Comparing neutrophil counts in the morning during TFL, we found that at day 5 during the TFL the total neutrophil count normalized overnight to baseline levels. After 7 days of cycling, an increased number of cells was detected after overnight recovery (1.26 fold-increase; 95%CI 1.01–1.51 p = 0.0431 (Table 4, Fig 2B). Thus, neutrophilia did not recover at the morning of day eight after a seven-day strenuous cycling tour.

**Table 3:** Evening peripheral blood neutrophil counts.

	Cell counts			
	day 0	follow-up	Mean difference	95% CI of difference
**Evening d0 vs. evening d4**	2.40	3.51	1.11	0.38–1.85^a^
**Evening d0 vs. evening d7**	2.40	3.67	1.26	0.44–2.09^a^

Cell counts in 10^6^ cells/ml

^a^ p <0.001

**Table 4:** Neutrophil subset counts.

	Cell counts			Mean difference		
	day 1	day 5	day 8	day 5 vs. day 1	day 8 vs. day 1	day 8 vs. day 5
**Total neutrophils**	1.78	1.89	2.24	0.11	0.46^a^	0.35
**CD16**^**bright**^**CD62L**^**bright**^	1.49	1.39	1.44	-0.11	-0.05	0.05
**CD16**^**bright**^**CD62L**^**dim**^	0.22	0.37	0.58	0.15^a^	0.36^b^	0.21^c^
**CD16**^**dim**^**CD62L**^**bright**^	0.02	0.05	0.07	25.8	0.04^c^	0.02

Cell counts in 10^6^ cells/ml

^a^ p <0.05

^b^ p<0.0001

^c^ p<0.005

### The maintained increases of neutrophil subsets counts were independent of changes in peripheral blood ACTH or cortisol levels

**Fig 3:**
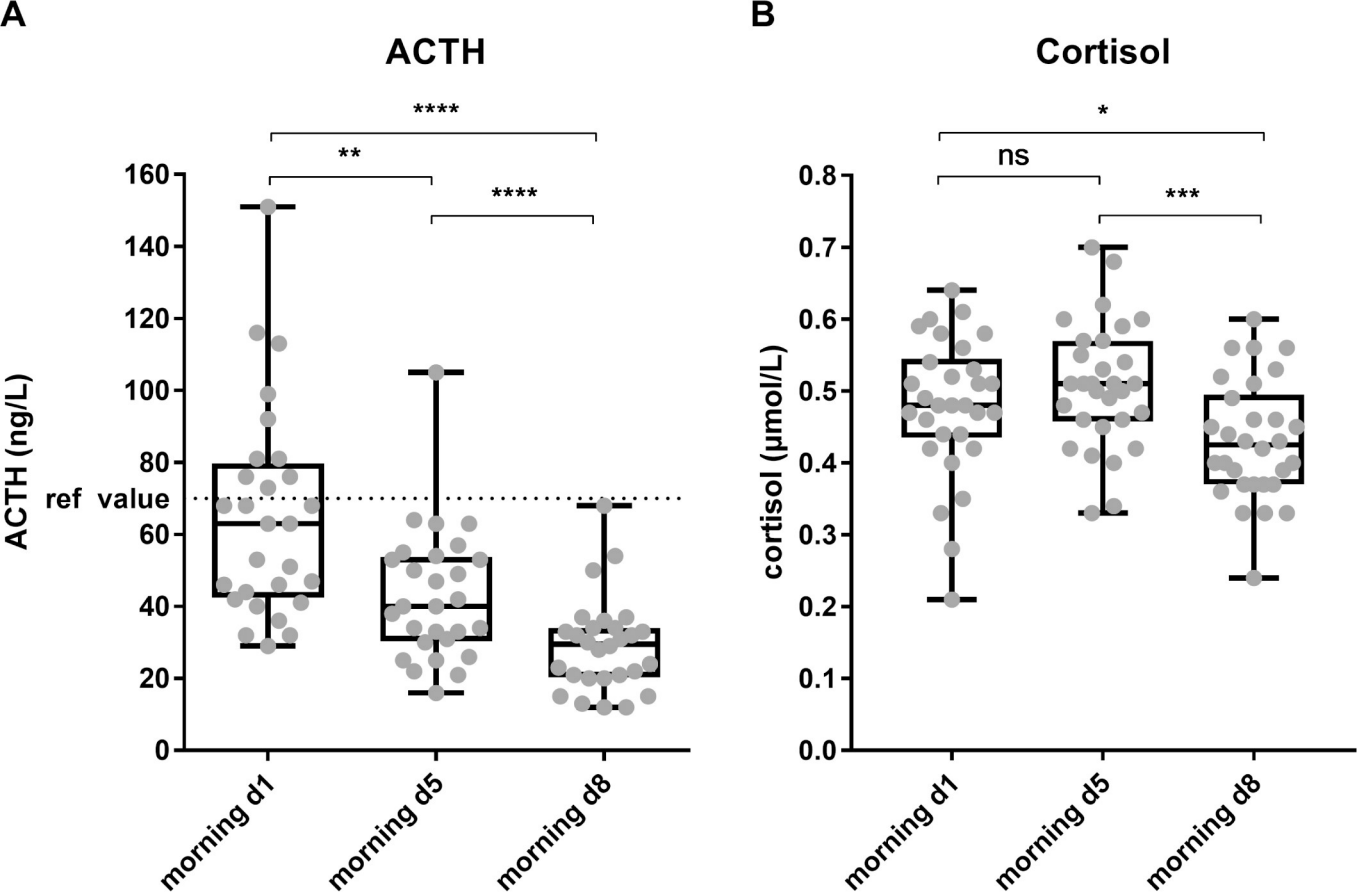
A decrease in ACTH plasma concentration was found over time, while serum cortisol concentrations showed a delayed decrease. A) Plasma ACTH concentrations in ng/L at morning day 1, day 5 and day 8 (n = 28). B) Serum cortisol concentrations at morning day 1, day 5 and day 8 are depicted in μmol/L (n = 29). * p = 0.0323; ** p<0.005; *** p<0.0005; **** p<0.0001.

To test the hypothesis that the maintained neutrophilia after prolonged exercise was caused by a rise in plasma ACTH and/or cortisol levels, we studied the relation between the increase in neutrophil counts and the ACTH and cortisol concentrations in the plasma. We measured these hormones at the same time point in the 3 mornings. Surprisingly, we found a clear decrease in ACTH plasma concentration during the TFL (Fig 3A). For cortisol there was no significant change in concentration halfway during the TFL, but levels were also significantly decreased at day 8 compared to day 1 (Fig 3B).

### Intensified endurance exercise led to mobilization of different neutrophil subsets

The finding that the impaired recovery of the innate immune response did not coincide with increased ACTH and/or cortisol levels implied that the maintained neutrophilia could not simply be explained by steroid-induced demargination of neutrophils into the circulation. To study whether the neutrophilia was caused by the mobilization of extra neutrophil subsets such as evoked by a systemic LPS challenge in healthy volunteers [16], we analyzed these cells in the context of their differential expression of CD16 and CD62L. During the TFL we collected blood samples and stored the leukocyte fractions by freezing. Once back in The Netherlands we thawed the cells and performed flow cytometry analysis as described by de Ruiter et al [29]. We confirmed that this method still enabled us to distinguish between the different neutrophil subsets (S2A Fig).

**Fig 4:**
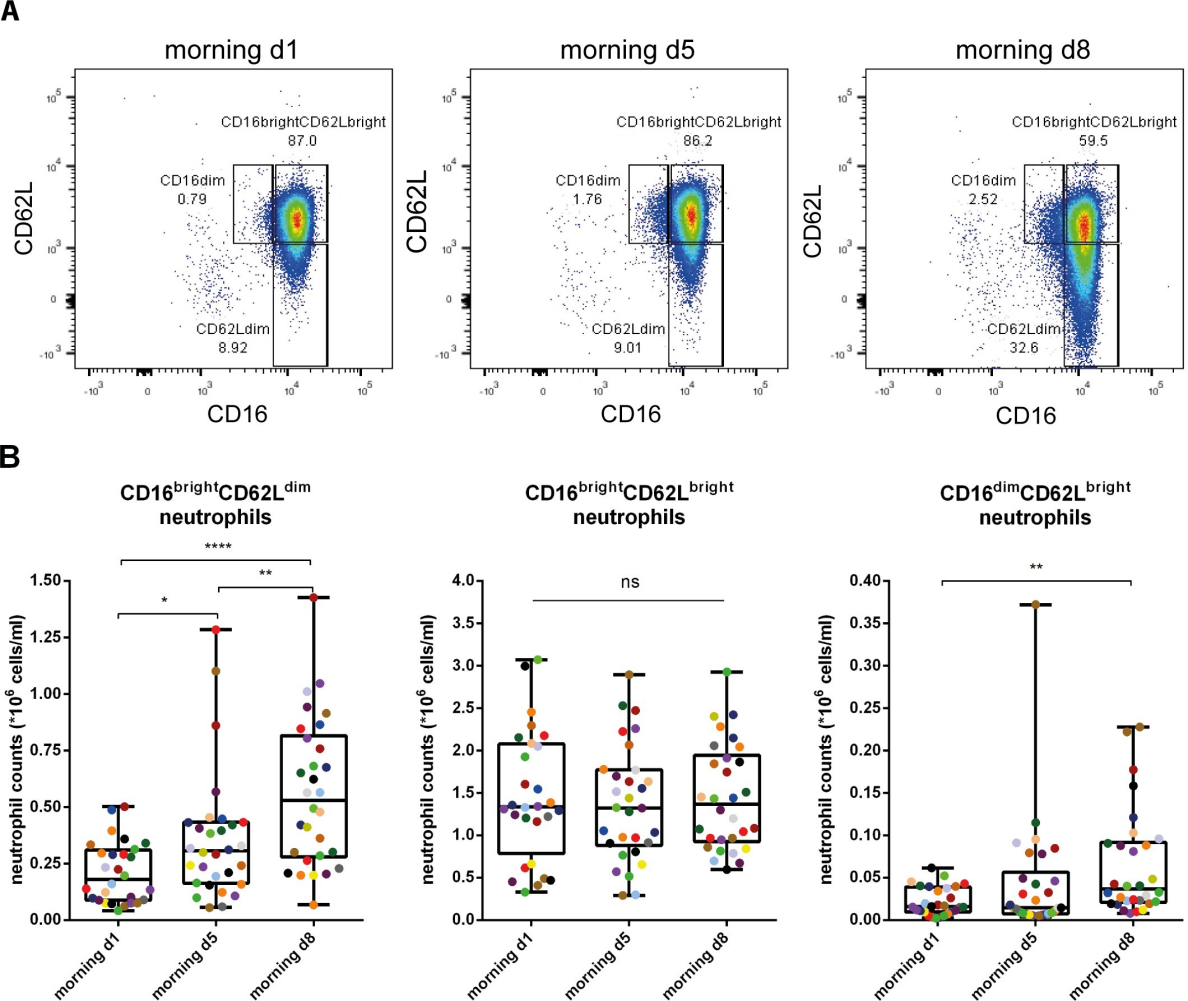
Increasing numbers of CD16^bright^CD62L^dim^ and CD16^dim^CD62L^bright^ neutrophils are found. A) Representative FACS plot of CD16 vs. CD62L expression of the total neutrophil population of one individual at the 3 morning time points during the TFL. B) graphs of the three CD16CD62L neutrophil subset counts at morning day 1, morning day 5 and morning day 8 per individual (n = 28). Colors visualize the individual changes over time. ns: not significant; * p<0.05; ** p<0.005; **** p<0.0001.

When analyzing the counts of the individual neutrophil subsets we found marked changes over time (Fig 4A). Normal segmented CD16^bright^CD62L^bright^ neutrophil counts remained stable over all morning time points (0.93 fold-increase (95% CI 0.65–1.19; p = 0.80) at day 5 and 0.96 fold-increase (95% CI 0.69–1.23; p = 0.95) at day 8 when compared to day 1), whereas CD16^bright^CD62L^dim^ neutrophils were significantly increased by 1.68 fold at morning day 5 (95% CI 1.04–2.39; p = 0.0376) and by 2.64 fold at morning day 8 (95% CI 1.95–3.37; p<0.0001) when compared to morning day 1. Also a 3.00 fold-increase (95% CI 1.45–4.55; p<0.005) in CD16^dim^CD62L^bright^ neutrophils was found at morning day 8 when compared to morning day 1 (Table 4, Fig 4B). Thus the sustained neutrophilia after exercise can be at least in part explained by the appearance of extra neutrophil subsets into the peripheral blood.

### Intensified endurance exercise led to complex changes in the phenotype of the different neutrophil subsets

**Fig 5:**
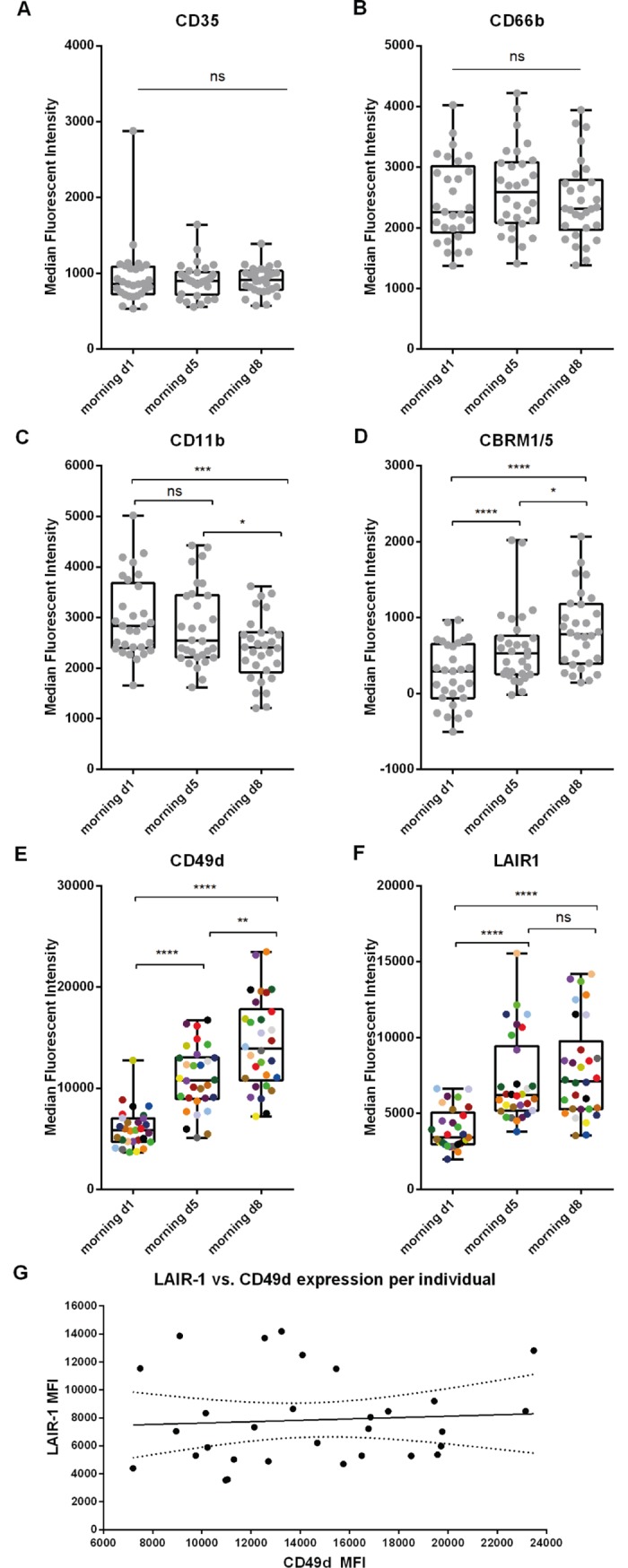
Intensified endurance exercise leads to a marked change in the peripheral blood neutrophil phenotype. Median fluorescent intensities for A) CD35, B) CD66b, C) CD11b, D) CBRM1/5, E) CD49d and F) LAIR-1 are depicted for all individuals at the 3 morning time points day 1, day 5 and day 8 (n = 30). Colors in E and F visualize the individual changes over time. G) Scatter plot of the MFI of CD49d at day 8 vs. the MFI of LAIR-1 at day 8 per participant (R^2^ = 0.0046, p = 0.7218), dotted lines represent the 95% CI. ns: not significant; * p<0.05; ** p<0.005; *** p<0.0005; **** p<0.0001.

Next to determination of the expression CD16 and CD62L to identify the different subsets, we studied the expression of the classical activation/degranulation markers CD35, CD66b and CD11b by flow cytometry. In addition, CD49d (very late antigen 4, VLA-4) and LAIR-1 (CD305) were included in the panel as markers for immature or alternatively activated neutrophils.[30–33] Of note, our freeze thaw method possibly permeabilizes granulocytes and, therefore, both extracellular and intracellular proteins might be measured. However, activation markers and their responsiveness to stimuli like fMLF could still be clearly measured.[29] Also, when employing this protocol on peripheral blood of LPS challenged volunteers, we did find differences in expression of the classical degranulation markers CD35 and CD11b between LPS subsets (S2B and S2C Fig), which was in line with previous results.[16] During the TFL, the classical activation/degranulation markers CD35 and CD66b were unaffected (Fig 5A and 5B). CD11b expression was lowered (Fig 5C), whereas the expression of CBRM1/5, an epitope expressed by CD11b in its activated state, significantly increased at day 5 (2.4 fold (95% CI 1.80–3.01) and at day 8 (3.3 fold (95% CI 2.57–4.03) (Fig 5D). The expression of CD49d increased markedly over the time course of the TFL from 1.80 fold-increase at day 5 (95% CI 1.57–2.04) to a 2.38 fold-increase at day 8 (95% CI 2.02–2.73) compared to day 1 (Fig 5E). The expression of LAIR-1 was increased by 1.82 fold (95% CI 1.53–2.10) halfway during the TFL and this increase was maintained at 2.00 fold (95% CI 1.68–2.33) at the end of the TFL when compared to morning day 1 (Fig 5F). The results of the statistical analysis are depicted in Table 5. At day 5 two groups are visible when analyzing LAIR-1 expression: part of the participants showed a steep upregulation of LAIR-1 expression, while the other participants upregulated LAIR-1 moderately. Most of the participants with high LAIR-1 expression at day 5 also highly expressed LAIR-1 at day 8. For CD49d we also found that most participants with high expression at day 5 maintained high expressions at day 8. However, no correlation was found between the MFI’s of LAIR-1 and CD49d (R^2^ = 0.0046, p = 0.7218, Fig 5G).

**Table 5:** Median Fluorescent Intensities of 6 neutrophil markers.

	MFI			MFI mean difference		
	day 1	day 5	day 8	day 5 vs. day 1	day 8 vs. day 1	day 8 vs. day 5
**CD35**	951	905	909	-47	-42	4
**CD66b**	2442	2638	2441	-196	1	198
**CD11b**	2989	2842	2380	-147	-609^a^	-462^b^
**CBRM1/5**	250	602	827	352^c^	576^c^	225^b^
**CD49d**	6039	10891	14346	4853^c^	8308^c^	3455^d^
**LAIR1**	3967	7209	7953	3242^c^	3986^c^	744

MFI’s are in arbitrary units

^a^ p <0.0005

^b^ p<0.05

^c^ p<0.0001

^d^ p<0.005

Since three different neutrophil subsets were found in the peripheral blood of the participants, the expression of above mentioned markers per neutrophil subset were also analyzed. Only small differences were found. The expressions expression of CD35 and CD49don CD16^dim^CD62L^bright^ neutrophils were significantly decreased when compared to mature CD16^bright^CD62L^bright^ neutrophils at morning day 1 and day 8, respectively. No other differences in expression were found between the subsets (S3 Fig).

### Multi-dimensional FLOOD analysis identified the exercise-induced changes in marker expressions that are found on the total population of peripheral blood neutrophils

**Fig 6:**
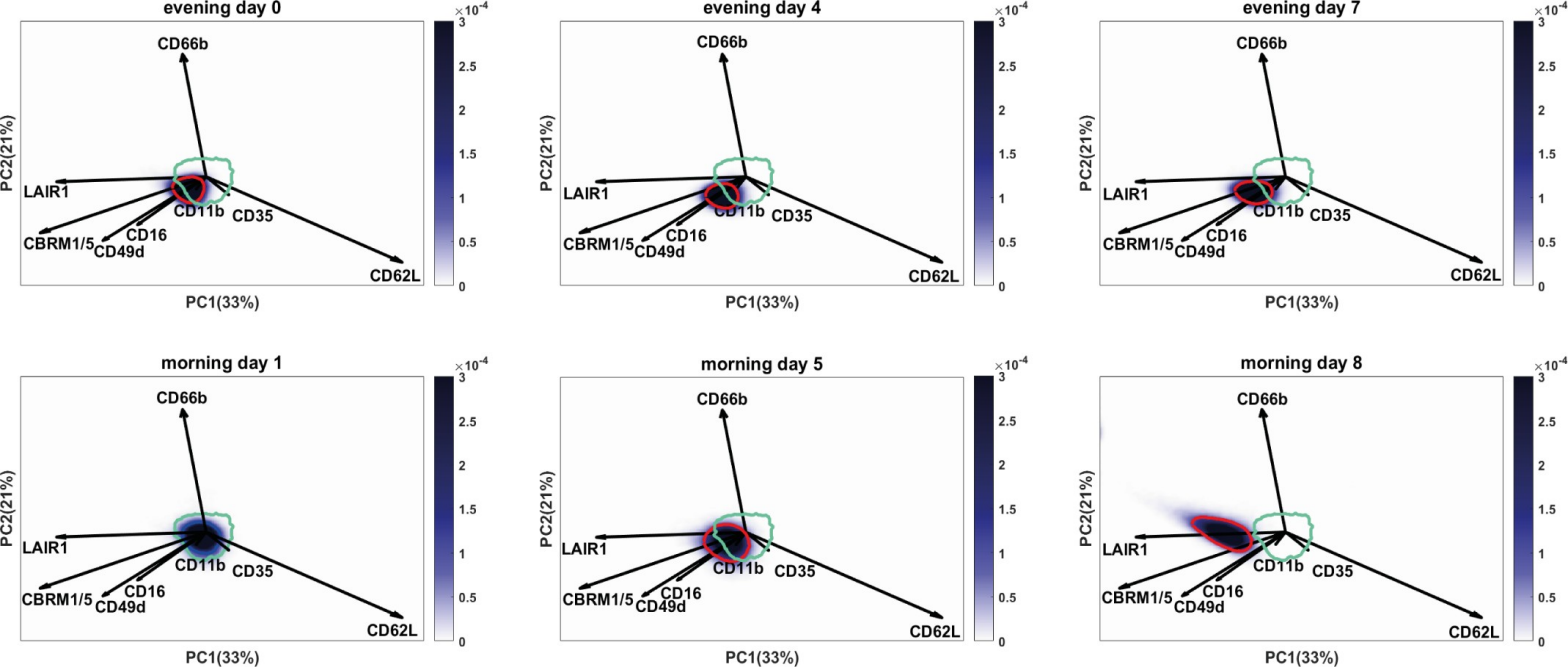
Multi-dimensional FLOOD analysis shows results found by conventional gating analyses in one biplot. FLOOD output: neutrophil deep phenotyping results at evening day 0, morning day 1, evening day 4, morning day 5, evening day 7 and morning day 8 in the multivariate response space. Results of six time point of one representative subject are shown here. Cells are depicted in a blue density gradient. The length of the vector of each marker correlates to the relative importance of that marker for the description of the multivariate space. The correlations between the different markers is defined by the angle between the vectors. Vectors pointing in the same direction are positively correlated; vectors in an angle 90° are not correlated with each other; vectors positioned in an angle of 180° are negatively correlated. The cyan circle depicts the region of 80% of the cells of all the control samples (day 1).The dark blue benchmark in the morning day 1 plot encompasses 80% of the cells of this specific sample. The neutrophils of this control sample fall within the cyan control benchmark. In the graphs of the other time points the red benchmark encompasses 80% of the cells of this individual. The cells shifted in the multivariate space towards CBRM1/5, LAIR-1, CD49d and CD16. Overnight from evening day 4 to morning day 5 the shift was maintained, but part of the cells still overlapped with the control benchmark. A total shift of the cells was seen at morning day 8, in the opposite direction of CD62L and more towards LAIR-1, CBRM1/5 and CD49d. Also a larger spread of the cells was found in the negative direction of CD62L.

The flow cytometry results so far were obtained by classically analyzing the data in a univariate or bivariate (CD16/CD62L) manner. We next evaluated whether these changes in marker expressions were correlated to each other. Moreover, we aimed to find out whether other neutrophil subsets than the CD16/CD62L subsets were responsible for the changes in MFI’s or whether the total neutrophil population exhibited up- or downregulation of the markers. To answer this question, we performed a computational Principal Component Analysis (PCA) based multivariate analysis: FLow Cytometric Orthogonal Orientation for Diagnosis (FLOOD) on the TFL data [25]. In the analysis the morning day 1 samples (control group) were compared to the evening day 0, evening day 4, morning day 5, evening day 7 and morning day 8 samples (defined as the response groups). In short, a response specific multi-dimensional model (‘response model’) was created in which the single cell variation within the response group was best explained. The FLOOD output consists of multi-dimensional plots (Fig 6) projected in 2D [25]. Per individual six plots were generated: one plot per time point. The plots contain data of one sample of a representative individual, and the data of all participants at morning day 1 (80% of the morning day 1 cells of all the participants are within the cyan benchmark). The data of the representative individual is encompassed by a blue benchmark at morning day 1 and by a red benchmark at evening day 0, evening day 4, morning day 5 evening day 7 and morning day 8. The relative importance of a marker for the description of the multi-dimensional space is depicted as the length of the vector. The relation in expression of the markers is depicted in the angle of the vectors [25].

For the TFL dataset 54% of the total response specific variance is shown in the PC1/PC2 plots (explaining 33% and 21% of the response specific variation, respectively). The results of the FLOOD analysis were as follows:

Firstly, an exercise induced shift of the peripheral blood neutrophil population was visible towards the direction of vectors of markers CBRM1/5, CD49d, LAIR-1 and CD16 (these vectors represent the PCA loadings). This was both found at evening day 4 as well as at evening day 7. Strikingly, the shift of the neutrophil population did not recover overnight. In fact, the shift was even more pronounced at morning day 8. At morning day 8 the shift was directed more clearly in the opposite direction of the CD62L loading vector, indicating that the cells were relatively more CD62L^dim^ when compared to the neutrophils at the previous time points. The loading of CD66b is orthogonally oriented to the loading vectors of LAIR-1, CBRM1/5, CD49d, CD16 and loadings CD62L and CD35. This implies there was no correlation between CD66b and the other markers. The CD16 loading vector is aligned with CD49d, CBRM1/5 and LAIR-1, but the length of the loading vector is relatively short, which indicates its minor importance for the variance explained by this model. The same accounts for CD11b and CD35, which have very short loading vectors. Therefore, CD11b, CD35 and CD16 did not contribute to the model. From the FLOOD plots one can appreciate that the shift of the whole cell population is quite dramatic: the cells at day 8 did not overlap with the region where the cells of the day 1 sample were found. This means that all the peripheral blood neutrophils changed their phenotype.

Secondly, besides a shift of the total neutrophil population, the cell populations at the morning time points have a larger surface area than the post-exercise cell population at evening day 4 and evening day 7. This indicates that the neutrophils at evening day 4 and evening day 7 were a more homogeneous cell population with less variance between the cells when compared to the morning time points. In addition, the total neutrophil population was more elongated in a diagonal direction at day 8. The elongation aligns to the CD62L loading, meaning that at day 8 the variance in CD62L expression was larger when compared to the previous time points. Together these findings corresponded well with what we found by univariate and bivariate analysis.

Thirdly, this analysis shows that the increase in CBRM1/5, LAIR-1 and CD49d expression was found on all neutrophils irrespective of their expression of CD16 and CD62L. After 4–7 days of intensive endurance exercise all neutrophils in the peripheral blood relatively express more LAIR-1, CBRM1/5 and CD49d. Overnight the neutrophil population did not recover and the cells were even more CD62L^dim^ at morning day 5 and day 8 (which in the example subject of Fig 6 is more prominent at day 8 when compared to day 5).

### The exercise induced neutrophil phenotype differs from the neutrophil subsets in LPS challenged volunteers

The flow cytometry results show both similarities and differences when compared to peripheral blood neutrophil phenotypes which arise during acute inflammation such as a evoked by LPS challenge [16]. We hypothesized that a long term exercise-induced neutrophil response, possibly driven by reperfusion problems and damage associated molecular patterns (DAMPs), would differ from a short term response to pathogen associated molecular patterns (PAMPs) such as LPS. To test this, we performed a second FLOOD analysis on both the TFL data as well as on data of samples obtained from LPS challenged volunteers at time point t = 180 minutes post LPS infusion. For both datasets the same multicolor flow cytometry panel was measured and all samples were processed using the same freeze thaw procedure [29]. In this analysis the TFL and LPS datasets were used to generate the response specific model to ensure that both the exercise response as well as the LPS response were described in the same response specific multi-dimensional space.

**Fig 7:**
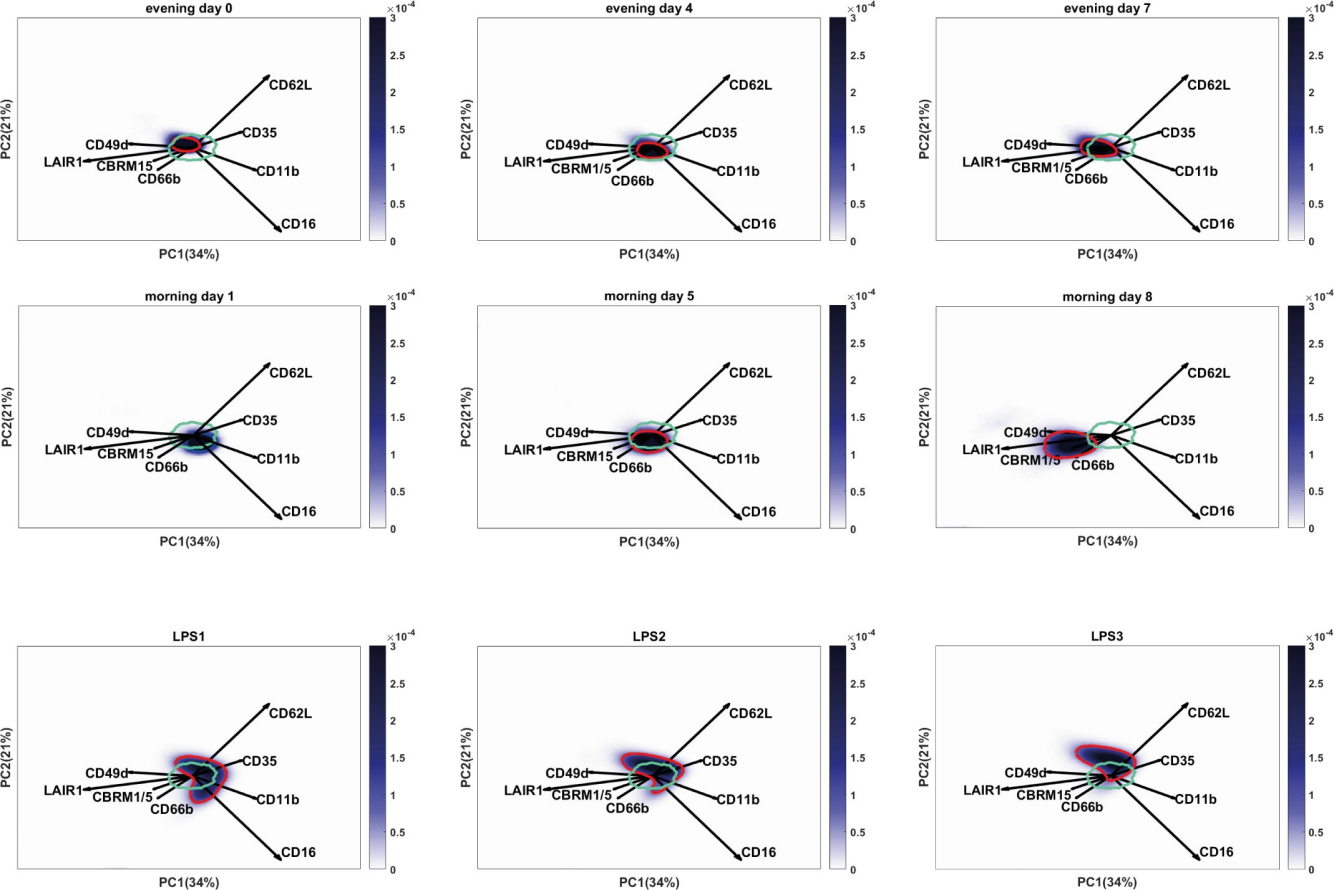
Exercise induced changes of neutrophil marker expressions differ greatly from peripheral blood neutrophil phenotypes found after LPS challenge. FLOOD analysis on TFL and LPS datasets. Cells are depicted in a blue density gradient. The cyan circle depicts the region of 80% of the cells of all the control samples (day 1). The dark blue benchmark in the morning day 1 plot encompasses 80% of the cells of this specific sample. The neutrophils of this control sample fall within the cyan control benchmark. In the graphs of the other time points the red benchmark encompasses 80% of the cells of this individual. A) During the TFL at the evening time points a shift towards LAIR-1, CD49d, CBRM1/5 and CD66b was found, negatively correlating with CD62L, CD35 and CD11b. B) The LPS samples showed a heterogeneous cell population with a CD16^dim^ subset and a CD62L^dim^ subset. A shift of the neutrophil cell population was found in the direction of loading vectors CD16, CD62L, CD35 and CD11b.

Fifty-five percent of the total response specific variance is described in the plots of Fig 7. For the TFL samples a shift is visible mainly towards LAIR-1 and to a lesser extent to CD49d, CBRM1/5 and CD66b (since the loading vectors are shorter). The shift is in the opposite direction of the loadings vectors of CD11b, CD62L and CD35, meaning these markers were downregulated. The shift of the cell population is orthogonally oriented to the loading vector for CD16, which indicates that CD16 did not contribute to the variance of the exercise induced neutrophil response.

The neutrophil phenotypes found in the peripheral blood 180 min after LPS infusion were clearly different from the exercise induced neutrophil phenotype, when comparing the location of the cells in the FLOOD plots. LPS samples showed the typical bean-shaped neutrophil population representing the different neutrophil subsets. The CD16^dim^CD62L^bright^ cells were found in the opposite direction of the CD16 loading; whereas the CD16^bright^CD62L^dim^ cells were found in the opposite direction of the CD62L loading and for this subset a higher expression of CD11b was found when compared to the other subsets. Compared to the TFL samples, the total LPS challenged neutrophil population upregulated CD35 and CD11b. Also a downregulation of CD16 and LAIR-1 on the total neutrophil population was found.

The TFL neutrophils at morning day 8 did not overlap with the region where the LPS neutrophils were found, meaning that the exercise-induced peripheral blood neutrophils exhibited a clearly different phenotype from LPS neutrophils.

## Discussion

In contrast to the numerous studies [24,34,35] investigating the innate immune response to a single exercise bout, our study examined the recovery of the neutrophil compartment at the morning of day 5 and 8 after daily intensified endurance exercise. We aimed to gain more insight into the mechanisms underlying the increased neutrophil counts in the peripheral blood during a prolonged and increased training load. We found that intensified endurance exercise led to impaired recovery of the innate immune response characterized by differences in activation markers and mobilization of different neutrophil subsets normally absent in the peripheral blood.

During the week of the TFL, amateur sports men and women increased their training volume on average with 770 ± 24%. Exercise induced neutrophilia recovered to baseline at the morning of day 5, but was significantly higher than baseline at the morning of day 8. This neutrophilia could not be explained by increases in serum cortisol and/ or plasma ACTH since these hormone levels were either stable or even decreasing in the mornings of the TFL. Other studies have reported stable resting cortisol levels after a period of intensified training [11–14] or even an increase in cortisol levels.[12] However, these studies enrolled professionally trained athletes instead of amateurs and increased the training load with 2.5 to 6 fold, that was less compared to our study.

Interestingly, in our study the ACTH levels did not relate to the cortisol levels nor to leukocyte numbers. This suggests that cortisol levels were either not controlled by ACTH or half-lives of both hormones were completely different. Similar dissociation of ACTH and cortisol has been described before in inflammatory mouse models.[36,37]

**Fig 8:**
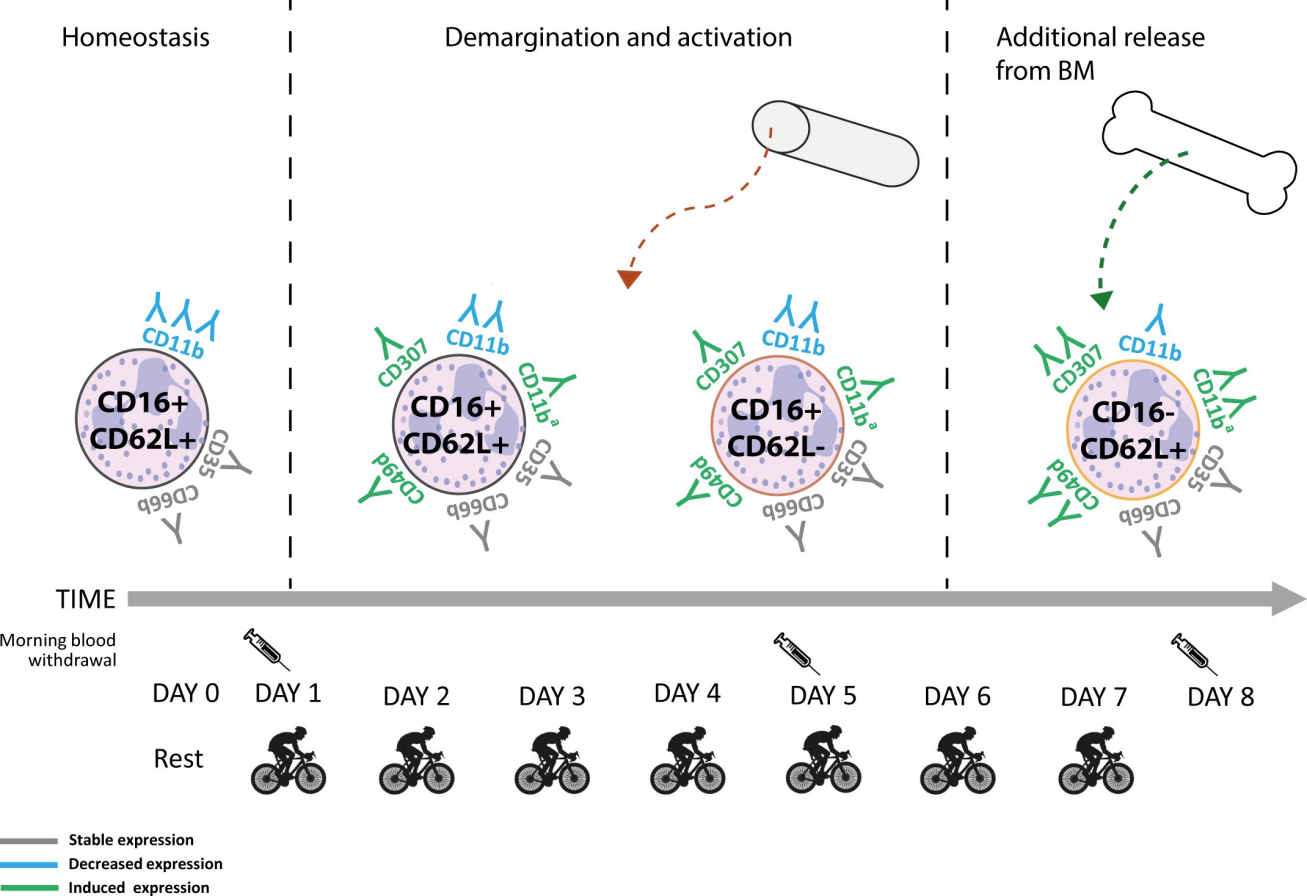
Schematic overview of neutrophil phenotypes emerging in the peripheral blood in the analysed time window. The various neutrophil subsets present in the peripheral blood at day 0, day 5 and day 8 are depicted based on their CD16 and CD62L expression. The downregulation of CD11b (in blue) and the upregulation of LAIR1, CD49d and CD11b^a^ (CBRM1/5, the activated epitope of CD11b) are visualised (in green) on the neutrophil cell membrane. The expression of CD35 and CD66b remained stable over time (in grey). At day 5 the rise in CD16+CD62L- neutrophils might be caused by demargination from the blood vessel wall, activation or mobilization from outside the vasculature. At day 8 an additional neutrophil subset with a CD16-CD62L+ phenotype was recruited from the bone marrow.

In order to study the characteristics of the neutrophilia and changes in the neutrophil compartment during recovery in more detail we performed multi-color flow cytometry. We applied a field applicable method that has recently been reported by us. [29] This method employs fixing and freezing and, therefore, the reported MFI’s in this article cannot be directly compared to studies employing fresh cells. However, irrespective of this limitation, our protocol allowed the identification of the two extra neutrophil subsets mobilized by LPS challenge (S2A Fig). Also, upregulation of CD11b and CD35 on CD16^bright^CD62L^dim^ cells when compared to CD16^dim^CD62L^bright^ cells was found similar (S2B and S2C Fig). This demonstrates the validity of the new protocol to identify subtle changes in phenotypes of white blood cells obtained from blood samples collected during field work. Similarly as found during acute inflammation evoked by LPS, two extra neutrophil subsets were mobilized during intensified endurance exercise. However, the phenotype of these two extra neutrophil populations is clearly different between induction by LPS and exercise. This is illustrated by the fact that no differences in CD11b and CD35 expression were found after 4 and 7 days of cycling between CD16^bright^CD62L^dim^ and CD16^dim^CD62L^bright^ neutrophils. Also the overall expression of markers on the complete neutrophil compartment was different as CBRM1/5, CD49d and LAIR-1 were clearly enhanced on all cells irrespective of the expression of CD62L and CD16 (see Fig 8 for a schematic overview of the different PMN phenotypes emerging in the peripheral blood over time). From this we can conclude that DAMPS liberated under conditions of exercise evoke an alternative response when compared to the PAMP LPS. Interestingly, the neutrophilia did not recover overnight to normal values after 7 days of cycling. This was particularly caused by the continued presence of the different neutrophil subsets in the peripheral blood and is in marked contrast to a single LPS challenge after which the blood neutrophil compartment completely normalized overnight.[38] Apparently, repeated intensified endurance exercise during the TFL evoked persistent signals leading to chronic mobilization of the different neutrophil subsets.

Importantly, the CD16^bright^CD62L^dim^ neutrophil subset has been described as immunosuppressive in an acute inflammation model by inhibiting T-cells.[16] In addition, these cells exhibit a low propensity to contain and or kill bacteria.[21] The occurrence of this subset in the blood and presumably also in the tissues during a period of intensified training might be part of an immune response to mild ischemia reperfusion injury.[39] The increase in neutrophil counts might be necessary for the repair of tissue damage [40] and the specific recruitment of this immunosuppressive neutrophil subset might be important for the inhibition of inflammatory responses to prevent collateral tissue damage, a key feature of reperfusion injury.[41] On the downside, these neutrophils would then respond inadequately to a potential infectious trigger by suppressing the function of other immune cells. This might contribute to the finding that profound exercise is associated with enhanced susceptibility for infections.

CD62L^dim^ neutrophils in the LPS model were characterized by a high expression of CD11b whereas the cells during the TFL showed lowered expressions, indicating important differences in these cell phenotypes between the two situations. In addition to the increase in CD16^bright^CD62L^dim^ neutrophils, we found both absolute and relative increases in CD16^dim^CD62L^bright^ neutrophils, at the morning of day 8. These cells were likely recruited from the bone marrow, mediated by an accelerated flux of cells through the neutrophil compartment such as proposed for inflammatory diseases.[42] Previous studies have reported increases in banded neutrophils upon endurance exercise.[18–20] The peripheral mature neutrophil pool (including the marginated and bone marrow pool) might become partly exhausted during 7 days of intensified endurance exercise as has also been described during leukocytosis induced by sepsis [43–45], leading to an increased release of banded cells from the bone marrow. Interestingly, we only found a clear increase in CD16^dim^CD62L^bright^ cells in 13 out of the 28 contestants at day 8. This finding cannot be explained by differences in age, training load or sex. We also found a heterogeneous response in expression of LAIR-1 and CD49d at day 8. This was found on all neutrophils irrespective of expression of CD16 and or CD62L. The unique heterogeneity in response to prolonged exercise may define novel leads to healthy training or even be used to individually monitor athletes in their pre-competitive training.

Next we analyzed MFI’s of several markers associated with neutrophil activation. Surprisingly, classical degranulation markers such as CD35 and CD66b did not show significant changes in any of the neutrophil phenotypes over time. The expression of the integrin CD11b was even downregulated over time. These data are in strong contrast to our findings during LPS challenge and clearly imply that neutrophils were not degranulating under these conditions in the peripheral blood. LAIR-1, CD49d, CBRM1/5 were found to be upregulated at day 5 and day 8 during the TFL. LAIR-1 is an immune inhibitory receptor binding to collagens and transducing negative signals into the cellular interior.[30,46,47] Therefore, it is tempting to speculate that the cells belong to a phenotype associated with immune suppression. The observed increase in CD49d and LAIR-1 might also be interpreted as the recruitment of (meta)myelocytes. However, this was unlikely as no distinct CD16^low^CD62L^low^CD66b^low^CD35^low^ subset was found during the TFL. Also the more immature CD16^dim^CD62L^bright^ cells did not express these markers differently from the CD16^bright^CD62L^bright^ cells. In fact, all three neutrophil subtypes similarly changed their expression profile of the aforementioned markers at day 8 of intensive endurance exercise, except from CD49d, which was downregulated in CD16^dim^CD62L^bright^ neutrophils at morning day 8 when compared to CD16^bright^CD62L^bright^ cells. However, even on the CD16^dim^CD62L^bright^ neutrophils CD49d expression was increased compared to morning day 1 and day 5. This supports the idea of a non-classical activation phenotype, rather than the rise of an immature neutrophil subset in the blood such as also has been described in sepsis patients.[48] Recently, also a circulating CD49d^bright^ pro-angiogenic neutrophil phenotype has been described in mice in response to hypoxia-induced VEGF-A signaling.[33] It is possible that the neutrophils present at day 5–8 after cycling upregulate their CD49d because they are exposed to both DAMPs originating from limited reperfusion as well as hypoxia signals.

The increased metabolic demand and anaerobic respiration caused by exercise lead to an increase in oxidative stress.[49] Generally this would result into the recruitment of activated neutrophils, potentially causing tissue damage such as seen in ischemia-reperfusion injury.[41] However, there were no clear indicators of reperfusion-associated damage during the prolonged period of intensified exercise during the TFL. Therefore, the increase of the CD49d^bright^LAIR1^bright^CBRM1/5^bright^CD11b^dim^ neutrophil subset is suggestive for an immunosuppressive neutrophil phenotype. It would be interesting to study the presence of such cells in patients who experience reperfusion injury such as found during coronary bypass surgeries, during ischemic stroke or in claudication patients.[50,51] In animal studies it has been proven that targeting the CD11b/CD18 (Mac-1) receptor on neutrophils is protective against reperfusion injury.[52,53] However, clinical trials in humans have been disappointing.[54,55] Future functional experiments focusing on the characteristics of exercise-induced neutrophils might tell us more about the pathways involved in neutrophil immunosuppression.

Besides this conventional flow cytometry analysis to identify cell populations and analyze MFI’s of several markers associated with neutrophil subsets and neutrophil activation, we also analyzed the data using the unsupervised multi-dimensional method FLOOD [25] to perform deep phenotyping of the data set. One of the benefits of this analysis is the 2D representation of the multiple correlations between all markers. This analysis is both more robust (no subjective gating) and much faster than the currently available multi-gating strategies. The FLOOD analysis for the TFL data clearly visualized an exercise-induced shift. The exercise-induced upregulation of CD49d, CBRM1/5 and LAIR-1 did not recover overnight. In fact, the shift was maintained at morning day 5 and even more pronounced at morning day 8 than directly post-exercise. From this we can conclude that the total neutrophil population did not normalize at day 5 and 8 after exercise.

Next to this, we compared the exercise induced neutrophil phenotype to the neutrophil subsets that arise upon LPS administration by analyzing both datasets together using the FLOOD method. From this analysis we can conclude an important finding: the exercise-induced activation of neutrophils differs greatly from the LPS (PAMP)-induced activation of neutrophils in the peripheral blood. The position of the TFL neutrophils at day 8 and the position of the LPS challenged neutrophils did not overlap with each other in the FLOOD response specific space, meaning none of these cells were the same.

This study is descriptive, but it provides us with new insights into the role of neutrophils during the changed innate immune response in response to periods of increased intensive endurance training. These changes might affect the normal immune response towards invading micro-organisms, which has been suggested by several studies (as reviewed in [56,57]). Regular physical exercise is correlated with fewer upper respiratory tract infections (URTI) when compared to a sedentary lifestyle.[58,59] On the other hand, periods of increased intensive endurance training in athletes are associated with an increased risk of URTI by yet poorly understood immunological mechanisms.[60] A single low-intensity exercise bout induces a biphasic mobilization of CD8+ T-cells and NK-cells. First the peripheral blood counts increase followed by a decrease resulting in lymphocytopenia.[61–63] High-intensity exercise has been found to lead to a diminished increase as well as a decrease of CD8+ T-cell counts,[64] which is hypothesized to create an “open window” [60] for pathogens like viruses to infect the host. Regardless, a systemic neutrophil response has been reported to precede the peak in CD8+ T-cell activation during a viral lung infection by 2–8 days [65] and might also be implicated in URTI after intensified exercise. These latter studies support the hypothesis of an important role of neutrophils in antiviral responses and could explain the increased risk of URTI during periods of intensified training in athletes. Our study is the first to show the maintained changes in neutrophil phenotypes upon intensified training even after overnight recovery. These changes may result in a more chronically impaired innate immune system, at least during periods of intensified training, supporting the “open window” hypothesis.[60]

In conclusion, at day 5 and 8 after intensified endurance exercise, the neutrophil compartment did not recover overnight. In fact, the aberrant neutrophil subtypes increased during the TFL. These neutrophils did not upregulate classical activation markers arguing against a degranulated phenotype. The observed changes in neutrophil phenotypes are in line with the hypothesis of immune suppression by neutrophils and could explain the increased risk of URTI during periods of intensified training in athletes. Our findings therefore provide a strong basis to design functional experiments to test this hypothesis.

## Supporting information

S1 Fig Neutrophil gating strategy. Neutrophils and monocytes were gated based on FSC and SSC, excluding eosinophils (large FSC/SSC), lymphocytes and debris. Single cells were gated. Monocytes were excluded from the neutrophil gate by gating the CD66b+ cells.
(TIF)

S2 Fig The neutrophil freezing protocol is sufficient for identification of neutrophil subsets based on CD16/CD62L expression. A) FACS plot of CD16 vs. CD62L expression of the total neutrophil population during acute inflammation (LPS model). Cells were frozen and thawed before staining the neutrophils. B) histogram of CD35 and CD11b expression of CD16^dim^CD62L^bright^ neutrophils (black) and CD16^bright^CD62L^dim^ neutrophils (gray) during acute inflammation C) histogram of CD11b expression of CD16^dim^CD62L^bright^ neutrophils (black) and CD16^bright^CD62L^dim^ neutrophils (gray) during acute inflammation.
(TIF)

S3 Fig Expressions of CD35, CD66b, CD11b, CBRM1/5, CD49s and LAIR-1 on the different CD16/CD62L neutrophil subsets. The median fluorescent intensities ± SD of A) CD35, B) CD66b, C) CD11b, D) CBRM1/5, E) CD49d and F) LAIR-1 are shown for the different neutrophil subsets at morning day 1, morning day 5 and morning day 8. * p<0.05.
(TIF)
