# Supplementary figures and images for: Multi-dimensional flow cytometry analysis reveals increasing changes in the systemic neutrophil compartment during seven consecutive days of endurance exercise
Source: PLoS One. 2018 Oct 30;13(10):e0206175. doi: 10.1371/journal.pone.0206175 (PMC6207321; doi:10.1371/journal.pone.0206175)

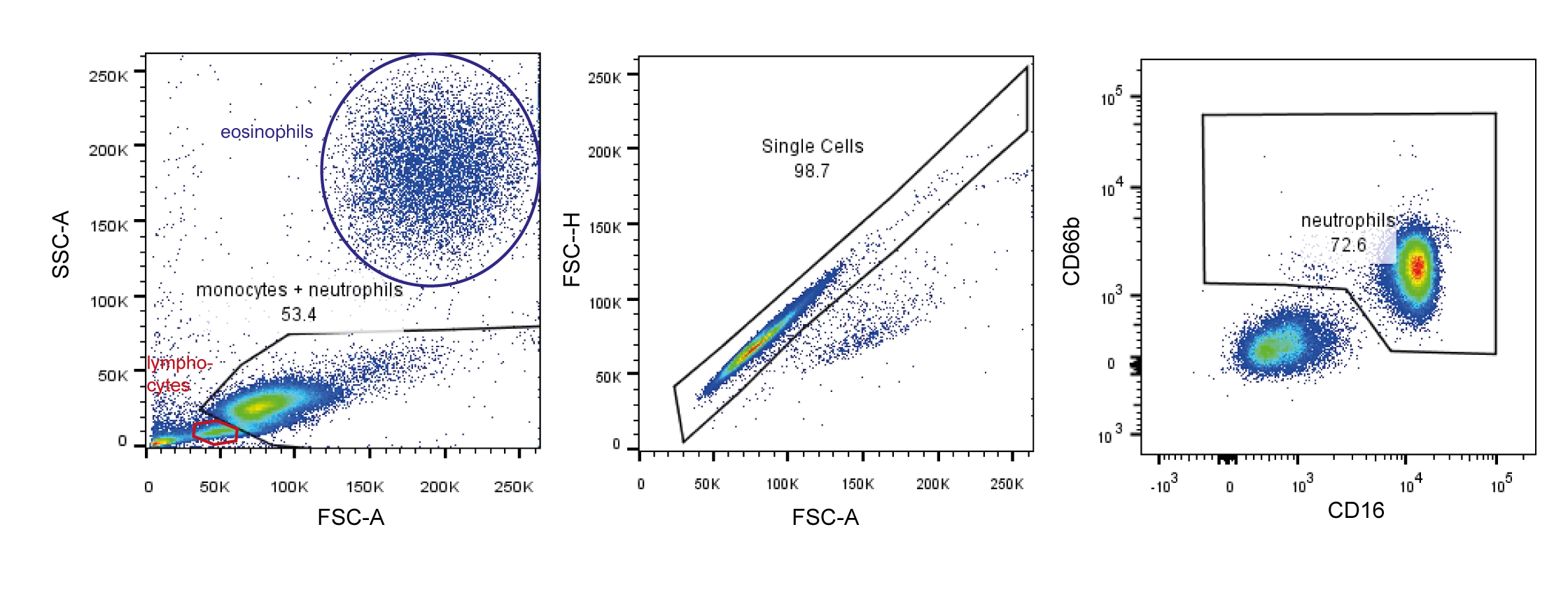

Supplement: S1 Fig — Neutrophils and monocytes were gated based on FSC and SSC, excluding eosinophils (large FSC/SSC), lymphocytes and debris. Single cells were gated. Monocytes were excluded from the neutrophil gate by gating the CD66b+ cells. (TIF) [file pone.0206175.s001.tif]

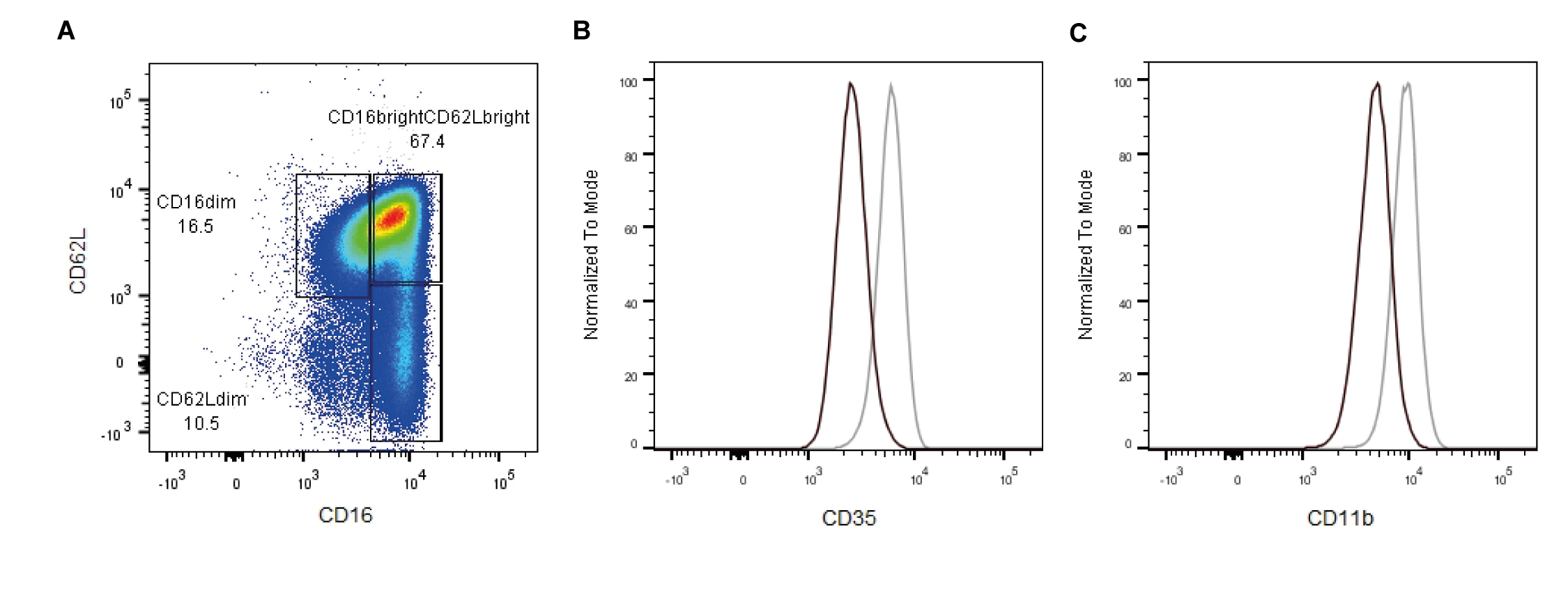

Supplement: S2 Fig — A) FACS plot of CD16 vs. CD62L expression of the total neutrophil population during acute inflammation (LPS model). Cells were frozen and thawed before staining the neutrophils. B) histogram of CD35 and CD11b expression of CD16dimCD62Lbright neutrophils (black) and CD16brightCD62Ldim neutrophils (gray) during acute inflammation C) histogram of CD11b expression of CD16dimCD62Lbright neutrophils (black) and CD16brightCD62Ldim neutrophils (gray) during acute inflammation. (TIF) [file pone.0206175.s002.tif]

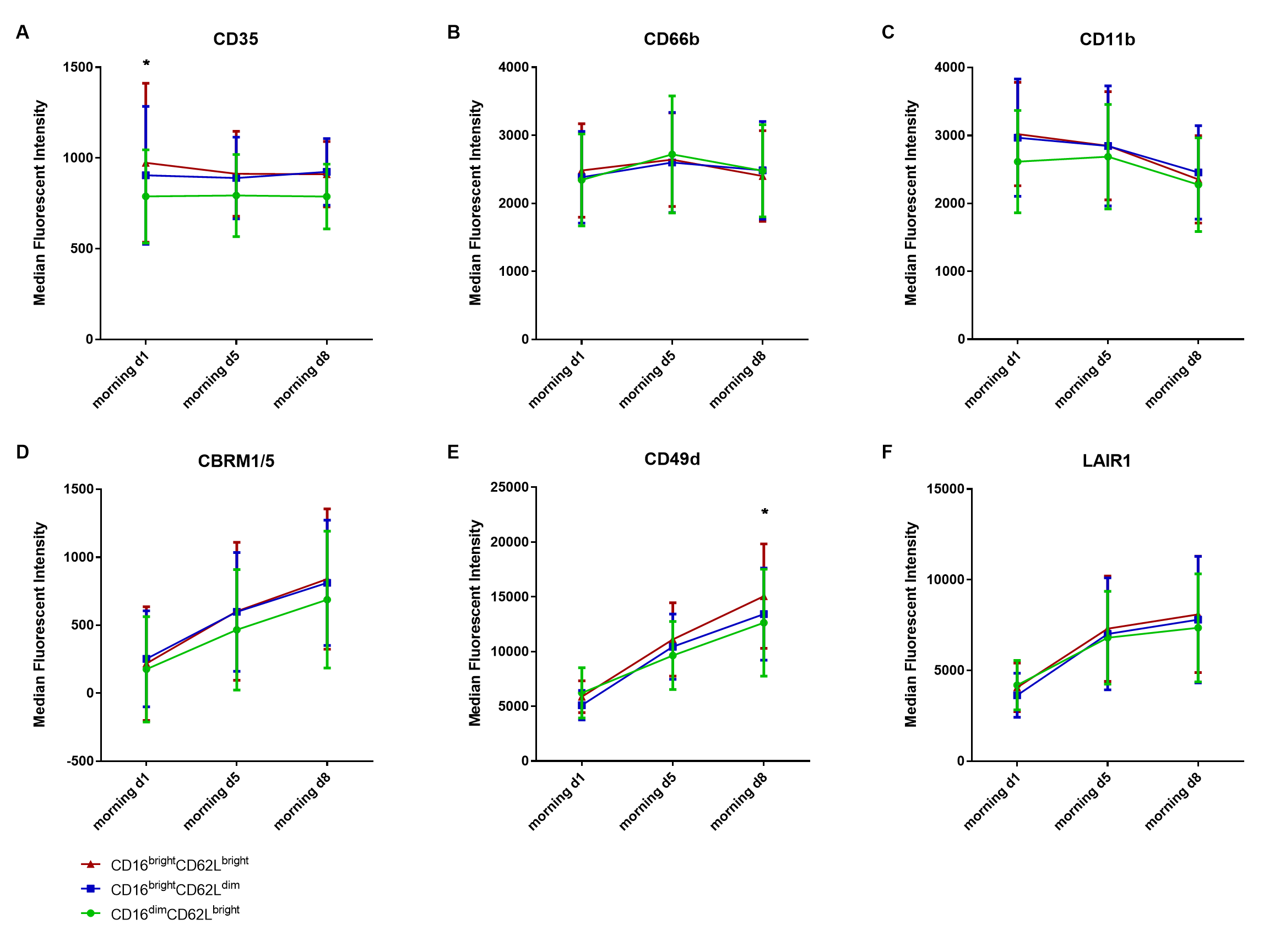

Supplement: S3 Fig — The median fluorescent intensities ± SD of A) CD35, B) CD66b, C) CD11b, D) CBRM1/5, E) CD49d and F) LAIR-1 are shown for the different neutrophil subsets at morning day 1, morning day 5 and morning day 8. * p<0.05. (TIF) [file pone.0206175.s003.tif]
